# Green Microalgae Strain Improvement for the Production of Sterols and Squalene

**DOI:** 10.3390/plants10081673

**Published:** 2021-08-13

**Authors:** Supakorn Potijun, Suparat Jaingam, Nuttha Sanevas, Srunya Vajrodaya, Anchalee Sirikhachornkit

**Affiliations:** 1 Department of Genetics, Faculty of Science, Kasetsart University, Bangkok 10900, Thailand; supakorn.pot@ku.th (S.P.); amp.jaingam@hotmail.com (S.J.); 2 Center for Advanced Studies in Tropical Natural Resources, National Research University-Kasetsart University (CASTNAR, NRU-KU), Kasetsart University, Bangkok 10900, Thailand; 3 Department of Botany, Faculty of Science, Kasetsart University, Bangkok 10900, Thailand; fscintsv@ku.ac.th (N.S.); fscisyv@ku.ac.th (S.V.)

**Keywords:** biodiesel, *Chlamydomonas*, squalene, sterol, terbinafine

## Abstract

Sterols and squalene are essential biomolecules required for the homeostasis of eukaryotic membrane permeability and fluidity. Both compounds have beneficial effects on human health. As the current sources of sterols and squalene are plant and shark oils, microalgae are suggested as more sustainable sources. Nonetheless, the high costs of production and processing still hinder the commercialization of algal cultivation. Strain improvement for higher product yield and tolerance to harsh environments is an attractive way to reduce costs. Being an intermediate in sterol synthesis, squalene is converted to squalene epoxide by squalene epoxidase. This step is inhibited by terbinafine, a commonly used antifungal drug. In yeasts, some terbinafine-resistant strains overproduced sterols, but similar microalgae strains have not been reported. Mutants that exhibit greater tolerance to terbinafine might accumulate increased sterols and squalene content, along with the ability to tolerate the drug and other stresses, which are beneficial for outdoor cultivation. To explore this possibility, terbinafine-resistant mutants were isolated in the model green microalga *Chlamydomonas reinhardtii* using UV mutagenesis. Three mutants were identified and all of them exhibited approximately 50 percent overproduction of sterols. Under terbinafine treatment, one of the mutants also accumulated around 50 percent higher levels of squalene. The higher accumulation of pigments and triacylglycerol were also observed. Along with resistance to terbinafine, this mutant also exhibited higher resistance to oxidative stress. Altogether, resistance to terbinafine can be used to screen for strains with increased levels of sterols or squalene in green microalgae without growth compromise.

## 1. Introduction

Sterols belong to a class of isoprenoid lipids found in the eukaryotic membrane. They perform crucial roles in maintaining membrane fluidity and permeability [1]. In animals, cholesterol is the major sterol that is essential for various biological processes. In fungi, ergosterol is the major type of sterol [2,3]. Sterols in plants or phytosterols perform similar functions as does cholesterol in terms of physiological functions and structure. Phytosterols have been clinically proven to be beneficial for human consumption [4]. They possess anti-cancer, anti-inflammatory, immunomodulatory and neuromodulatory properties [5,6,7]. The consumption of phytosterols have been shown to lower blood LDL-cholesterol levels and decrease the chance of developing a number of diseases [8,9]. The current source of phytosterols is from plant oil. However, due to the rising demand for phytosterols, microalgae have been suggested as an alternative source, as algal oils contain equal or greater amounts of sterols compared to plant oils [9,10]. Moreover, some microalgae also synthesize certain sterols such as 7-dehydroporiferasterol, that are not present in plants, which has been shown to possess outstanding anti-inflammatory activities [11].

**Figure 1:**
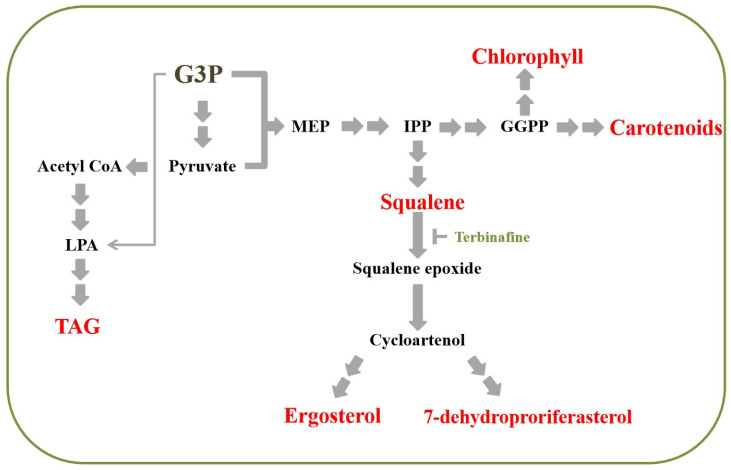
Simplified pathways for sterol, pigment, and triacylglycerol synthesis (G3P; glycerol-3-phosphate; GGPP: geranylgeranyl pyrophosphate; IPP: isopentenyl diphosphate; LPA: lysophosphatidic acid; MEP: 2-C-methyl-d-erythritol 4-phosphate; TAG: triacylglycerol).

The synthesis of sterols starts with isopentenyl diphosphate (IPP), a five-carbon compound that acts as a building block for all isoprenoids (Figure 1). In plants and algae, IPP can be derived from both the mevalonate (MVA) pathway, and the non-mevalonate or the methyl-D-erythritol 4-phosphate (MEP) pathway [12]. In green algae, however, the MVA had been lost, leaving the MEP pathway as the sole pathway for isoprenoid synthesis [13,14]. The first step of sterol synthesis in green algae is the production of squalene. Squalene is widely used in the pharmaceutical and cosmetics industries for its ability to hold moisture, its antioxidant and anticancer activities, and its protective effect against cardiovascular diseases [15,16]. It is also commonly used as an ingredient in vaccine adjuvants [17]. Currently, the major sources of squalene are plant oil and shark liver oil. Due to these unsustainable sources, microorganisms including microalgae have also been explored for the commercial production of squalene [18].

The cultivation of microalgae has been widely suggested as a way of utilizing light energy from sunlight for the production of high value compounds, and simultaneously reducing atmospheric carbon dioxide via photosynthesis [19]. As algae can utilize organic matter from industrial water for growth, the production of natural products could be combined with wastewater treatment. Nevertheless, several limitations such as the cost of cultivation, cell harvesting, and compound extraction still exist. Outdoor cultivation is also faced with several obstacles such as high light intensity and fluctuating temperature, both of which cause oxidative stress to the cell, lowering the biomass yield. Contamination and invasion by other algal species are also common [20,21]. Therefore, algal strains with higher resistance to abiotic stress, or being able to grow in conditions which others cannot tolerate would be favorable. Improving selected strains to increase yield of high value products is another direct approach to boost production efficiency [22].

Terbinafine is an antifungal drug that prevents the accumulation of sterols, leading to cell death [23]. It inhibits the enzyme squalene epoxidase, which converts squalene to squalene epoxide (Figure 1). Terbinafine was reported to interact with the enzyme, preventing the natural substrate from binding to the enzyme’s active site [24]. It is possible that greater tolerance to this drug might be a result of greater ability to generate more substrate molecules or better activity of the enzyme to bind with its substrate, squalene, which ultimately leads to overproduction of sterols. Other than resistance to this drug, resistant strains might have a higher tolerance to other stresses that microalgae might face in an outdoor cultivation setting. To explore this idea, UV mutagenesis was employed to generate a mutant population of the model green microalga *Chlamydomonas reinhardtii.* Three terbinafine-resistant mutants were successfully isolated. Physiological characterization of the mutants was performed and discussed.

## 2. Results and Discussion

### 2.1. Mutant Isolation and the Determination of Sterols, Squalene, and Pigment Content

**Figure 2:**
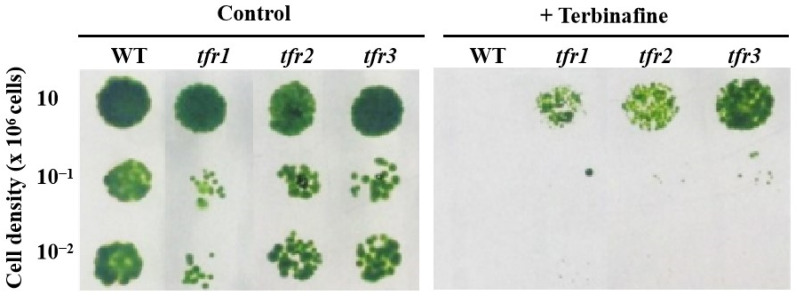
Growth phenotype of wild type (WT) and *tfr* mutants of *C. reinhardtii*. Serial dilutions of cells were spotted onto the agar medium without terbinafine (**left**) and with 1 mM terbinafine (**right**) and cultivated for 1 week.

**Figure 3:**
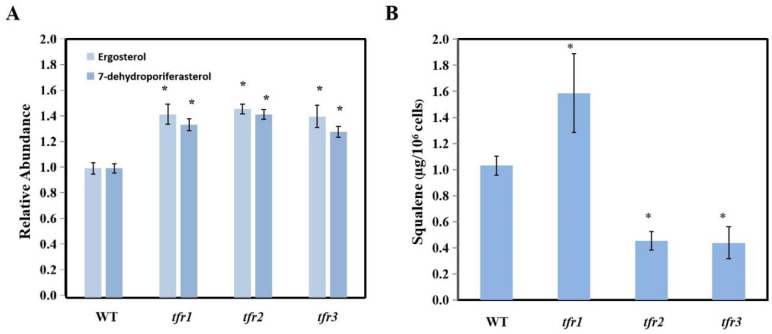
(**A**) Sterols and (**B**) squalene content. Total lipid was extracted from log-phase cultures. All data are means ± SD of three biological replicates. Significant differences between the wild type (WT) and the mutants within the same condition are indicated by asterisks (*) (*p* < 0.05).

The inhibition of intermediate steps in the sterol biosynthesis decreased the accumulation of sterol in plants and algae [25,26,27,28]. Cells that are able to resist inhibitors in the sterol biosynthesis might then have an upregulation of a certain step in the pathway, or a change in the activity of certain enzymes, leading to sterol accumulation. A UV-mutagenized mutant population of 1920 colonies was generated in the green alga *C. reinhardii*. They were tested on plates containing different concentrations of terbinafine. Three terbinafine-sensitive strains were isolated and characterized in our previously published work [29]. From this same mutant population, a total of three mutants were confirmed for their resistance to the drug (Figure 2). They were named terbinafine-resistant mutants, *tfr1*, *tfr2*, and *tfr3*. To study the effects of terbinafine resistance on the sterol content, the predominant sterols in *Chlamydomonas*, ergosterol and 7-dehydroporiferasterol, were analyzed using the GC-MS method. All three mutants exhibited an increase of approximately 50% in the level of both sterols compared to the WT (Figure 3A). In the presence of terbinafine, which targets the squalene epoxidase enzyme, squalene accumulation can be observed in this alga. The *tfr1* mutant accumulated 50% higher squalene level, whereas *tfr2* and *tfr3* showed 50% lower squalene level compared to that of the WT (Figure 3B).

**Figure 4:**
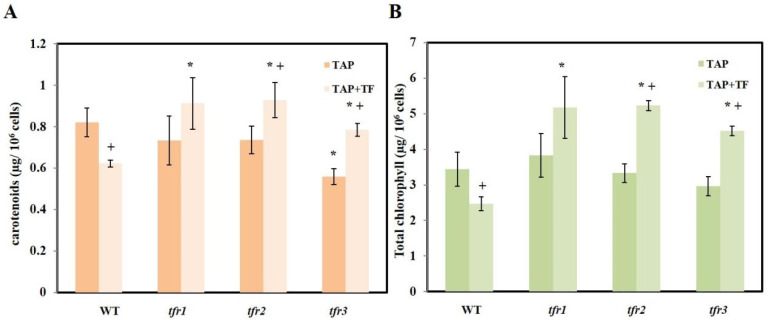
(**A**) Carotenoid and (**B**) chlorophyll content under N-replete medium in the presence and the absence of terbinafine. All data are means ± SD of three biological replicates. Significant differences between the wild type (WT) and the mutants within the same condition are indicated by asterisks (*) (*p* < 0.05). Significant differences between different treatments of the same strain are indicated by plus sign (+) (*p* < 0.05).

Chlorophyll and carotenoids are major photosynthetic pigments that are essential for photoautotrophic growth. Their synthesis also uses IPP as a precursor (Figure 1). Carotenoids also play a crucial role as antioxidants, preventing cell damage due to oxidative stress [30]. To investigate the effects of increased sterol content on pigment content, both chlorophyll and carotenoid levels were measured in all strains, with and without terbinafine being added. In normal medium, the levels of both carotenoid and chlorophyll were similar in all strains (Figure 4A,B). The only exception was the carotenoid level of *tfr3* that was slightly lower than that of the others (Figure 4A). Terbinafine treatment resulted in a decrease in both carotenoid and chlorophyll levels in the WT (Figure 4A,B). In contrast, both pigments showed elevated levels in all three mutants when terbinafine was added (Figure 4A,B).

The overaccumulation of sterols in *Chlamydomonas* terbinafine-resistant mutants is in agreement with previous reports in yeast. The yeast *Saccharomyces cerevisiae* and *Schizosaccharomyces pombe* terbinafine resistance mutants also exhibited higher ergosterol content than the wild-type cells [31,32]. Although the three mutants isolated in this work accumulated higher levels of sterol, only *tfr1* accumulated higher level of squalene, whereas the other two mutants exhibited only half of the WT squalene level. This difference could be due to the differences in their mutations. The increase of both ergosterol and 7-dehydroporiferasterol was similar in all mutants. As there was no change in the ratio of these sterols, the mutations were unlikely to reside in the later steps of the pathway. The mutation in *tfr1* might affect earlier steps, prior to squalene synthesis. An upregulation of the entire sterol pathway could lead to higher levels of intermediates upstream of squalene. By having more precursors for squalene synthesis, when squalene epoxidase enzyme is inhibited by terbinafine, this would result in a high level of squalene. On the other hand, the *tfr2* and *tfr3* mutants might have a desensitized squalene epoxidase or have high levels of the squalene epoxidase enzyme. The enzymes in the two mutants were able to convert more squalene into sterols, resulting in low squalene levels under terbinafine treatment. Nevertheless, evidence from the *Schizosaccharomyces pombe* mutant collection revealed that the mutations leading to terbinafine resistant phenotype could be in pathways other than squalene synthesis such as genes for mitochondrial function, ubiquitination, membrane trafficking, cell polarity, chromatin remodeling, and unknown genes [31].

Our previous work on terbinafine-sensitive mutants suggests that sensitivity to terbinafine could be used to screen for mutants that accumulate higher levels of triacylglycerol and carotenoids [29]. The mutants will be particularly useful for bringing microalgal biodiesel production closer to commercialization. All of the sensitive mutants, however, did not have higher levels of sterol. On the contrary, all of the terbinafine-resistant mutants in this work were found to produce higher levels of sterol. Therefore, it was clear that these two groups of mutants were affected in different pathways and could be used specifically for different purposes.

### 2.2. Effect of Environmental Stresses on Neutral Lipid and Pigments

**Figure 5:**
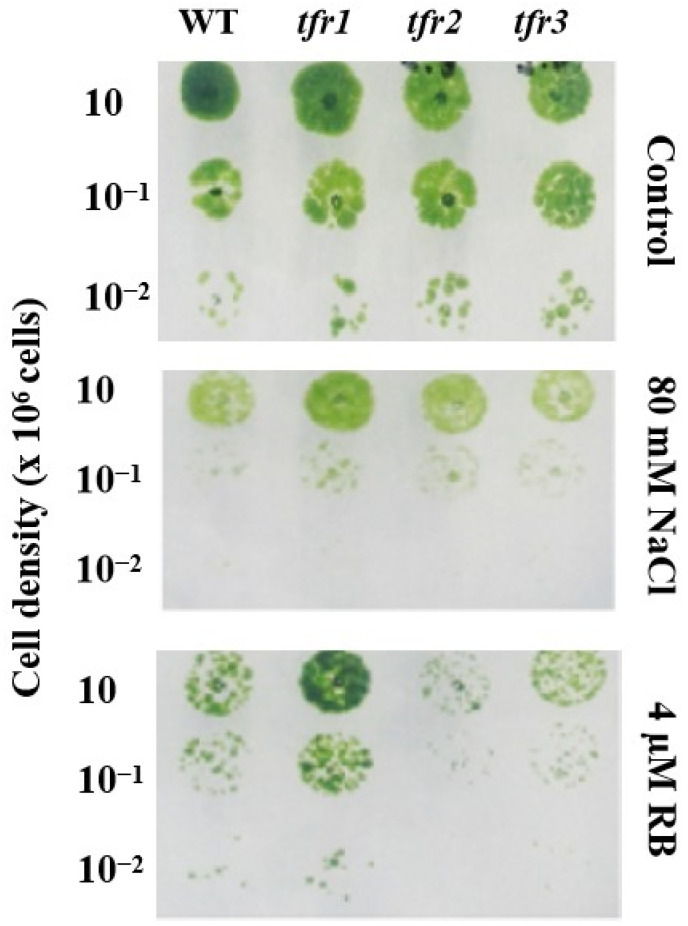
Growth phenotype wild type (WT) and *tfr1* under oxidative stress. Serial dilutions of cells were spotted onto the agar medium NaCl and Rose Bengal (RB) and cultivated for 1 week.

Cultivation of microalgae can be performed using water that is unsuitable for agriculture use such as brackish or industrial wastewater. Contaminants from wastewater and other environmental factors such as high light intensity, are known to induce oxidative stress [33,34]. In fact, tolerance to oxidative stress is a desirable property for algae grown in wastewater [35]. To investigate the phenotype of the WT and mutants under oxidative stress, growth on a TAP medium containing 80 mM NaCl and 4 μM Rose Bengal, a chemical that generates singlet oxygen, was tested. Only the *tfr1* mutant exhibited greater tolerance to these chemicals compared to other strains (Figure 5).

**Figure 6:**
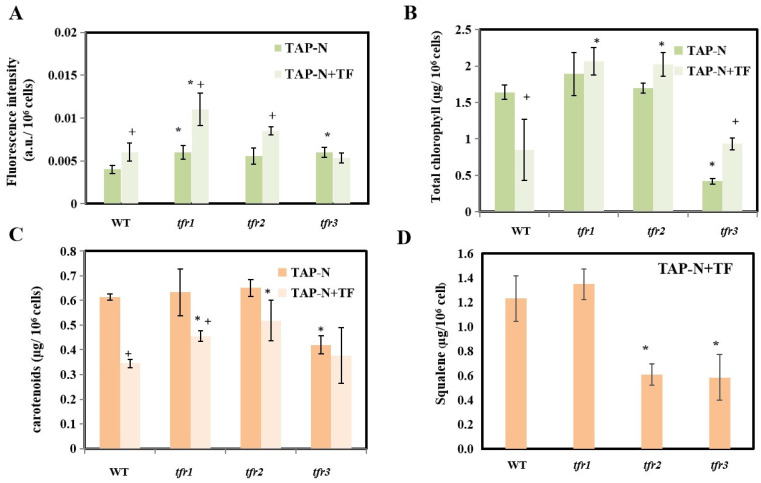
Quantification of triacylglycerol (**A**), chlorophyll (**B**), carotenoid (**C**) and squalene (**D**) from cultures under N-deprived condition with and without terbinafine. All data are means ± SD of three biological replicates. Significant differences between the wild type (WT) and the mutants within the same condition are indicated by asterisks (*) (*p* < 0.05). Significant differences between different treatments of the same strain are indicated by plus sign (+) (*p* < 0.05).

Other than oxidative stress, nutrient stress is another factor that algae face in outdoor cultivation [36]. As microalgae have been recognized for their biotechnological potential in the production of biofuels in the past decade, many studies have reported how stresses, especially nutrient stress, lead to increased lipid accumulation [37,38,39,40]. Triacylglycerol or TAG is the type of lipid that can be converted to biodiesel via trans-esterification. The nitrogen-deprivation stress is one of the most widely used conditions for inducing the accumulation of TAG [33,41]. To investigate changes in lipid production after nitrogen deprivation, all strains were placed in a nitrogen-depleted medium with and without terbinafine. Nile Red dye was used to quantify neutral lipids based on the degree of fluorescence in each sample. The presence of terbinafine led to an increase in TAG content in all strains with the exception of *tfr3* (Figure 6A). Interestingly, nitrogen starvation combined with terbinafine caused a great increase in TAG production in *tfr1* to about 2-fold compared to the WT level. In the case of pigments, the presence of terbinafine caused a decrease in the levels of both chlorophyll and carotenoids in the WT (Figure 6B,C). Under terbinafine treatment, the levels of both pigments in *tfr1* and *tfr2* were significantly higher than in WT, as opposed to *tfr3* that had lower levels of both pigments. For squalene, under nitrogen deficiency combined with terbinafine conditions, *tfr1* and WT accumulated similar level of squalene (Figure 6D). On the other hand, *tfr2* and *tfr3* still accumulated only 50% of the WT squalene level (Figure 6D).

Microalgae cultivated under environmental stress conditions such as nitrogen limitation, phosphorus deficiency, and high light intensity, are known to alter their lipid biosynthetic pathways towards the formation and accumulation of neutral lipids, mainly in the form of TAG [42,43]. The nitrogen starvation condition influences the distribution of carbon flux in microalgae [44]. Under this condition, degradation of the nitrogenous compounds such as proteins occurs, and carbon is diverted to storage compounds including lipids and carbohydrates [45]. The most interesting mutant was the *tfr1* strain for its tolerance to environmental stresses and due to the high accumulation of TAG (Figure 5 and Figure 6A). The TAG level was also elevated in the presence of terbinafine, which induced an accumulation of squalene. Cells under terbinafine treatment also contained more carotenoids than the WT. These phenotypes supported the idea that the mutation in this mutant might lead to an upregulation of the sterol synthesis earlier in the pathway. When terbinafine was used to inhibit sterol synthesis, there might be higher levels of intermediates available for both TAG and carotenoid synthesis. As sterols, squalene, and carotenoids possess antioxidant activities, the *tfr1* was also able to withstand stresses better than other strains.

### 2.3. Growth and Photosynthetic Efficiency

**Figure 7:**
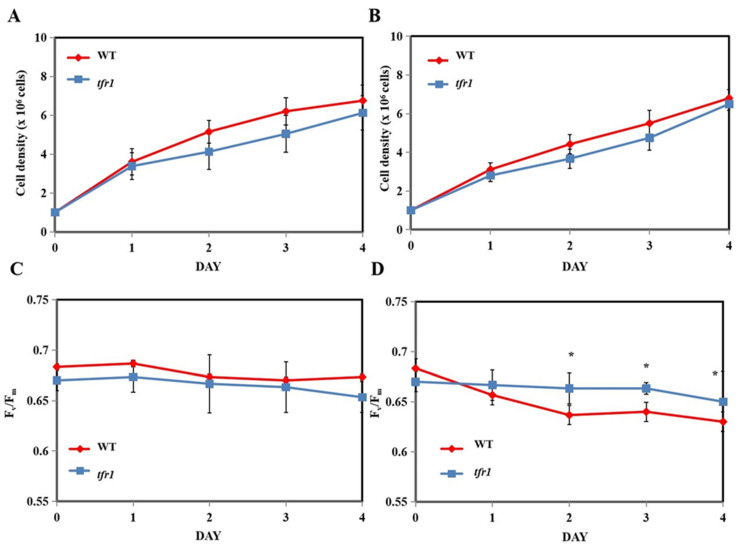
Growth and maximum quantum yield (F_v_/F_m_) parameter of wild type (WT) and *tfr1* mutant in nitrogen-replete medium (**A**,**C**) and nitrogen-replete medium with terbinafine (**B**,**D**). All data are means ± SD of three biological replicates. Significant differences between the wild type (WT) and the mutants within the same condition are indicated by asterisks (*) (*p* < 0.05).

**Figure 8:**
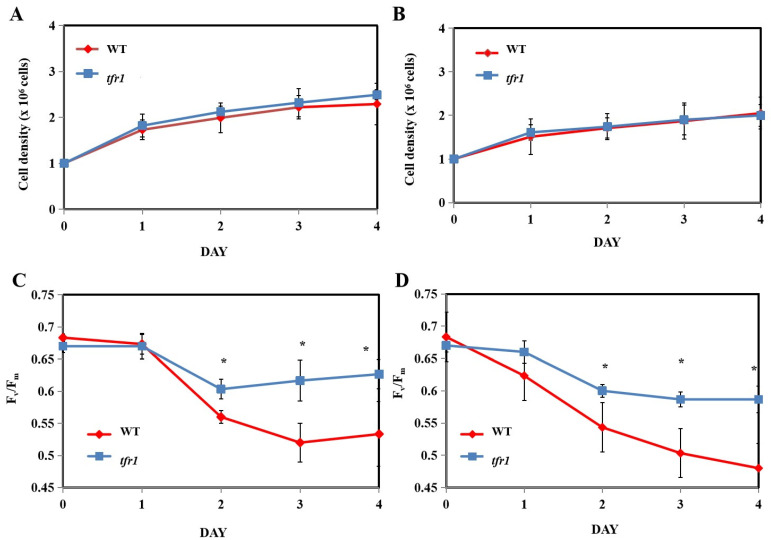
Growth and maximum quantum yield (F_v_/F_m_) parameter of wild type (WT) and *tfr1* mutant in nitrogen-depleted medium (**A**,**C**) and nitrogen-depleted medium with terbinafine (**B**,**D**). All data are means ± SD of three biological replicates. Significant differences between the wild type (WT) and the mutants within the same condition are indicated by asterisks (*) (*p* < 0.05).

As *tfr1* exhibited the most promising phenotype, it was further investigated whether or not the mutation with regard to this mutant affected the efficiency of photosynthesis and growth, both of which will affect product yield. Under nitrogen-replete cultivation conditions, there was no significant difference in cell growth between the WT and *tfr1*, with or without terbinafine treatment (Figure 7A,B). Light utilization needs to be efficient in order to maximize algal growth, especially in outdoor conditions, where the light intensity changes constantly. The maximum quantum yield (F_v_/F_m_) of photosystem II (PSII) can be used to monitor the efficiency of light utilization by the photosystem II (PSII) to estimate algal health [46]. There was essentially no difference in the F_v_/F_m_ values between the two strains when no terbinafine was added (Figure 7C). In the presence of terbinafine, however, the mutants exhibited higher F_v_/F_m_ values than the WT, especially on later days (Figure 7D). Similarly, under nitrogen starvation conditions, there was no difference in growth between the WT and the *tfr1* mutant (Figure 8A,B). However, unlike nitrogen-replete condition, nitrogen deprivation caused a substantial decrease in the F_v_/F_m_ values that never fully recovered to the initial values, especially in the case of WT (Figure 8C,D). The values of WT cultivated under nitrogen starvation conditions were lower than those of the mutant (Figure 8C). A great drop in the WT F_v_/F_m_ values was observed under the combination of nitrogen starvation and terbinafine, whereas the values of the mutant exhibited only a small drop (Figure 8D). Although a significant decrease in F_v_/F_m_ values was observed especially under nitrogen depletion condition, it does not directly translate to the same degree of growth inhibition. This is because F_v_/F_m_ measurement is rather sensitive for determining the efficiency of PSII functions. Any decline in F_v_/F_m_ values can be detected within hours of stress treatments. The growth condition used here was mixotrophic growth. Therefore, cells did not rely solely on photosynthesis. They were still able to propagate using the carbon source provided in the medium. As nitrogen starvation is known to induce oxidative stress, and the accumulation of TAG has also been suggested as a way to relieve this stress by acting as an electron sink [47,48], the *tfr1*, with the ability to produce more TAG, was able to better tolerate this condition, especially with the addition of terbinafine.

The *tfr1* strain is a great candidate for the development of high value lipid production. The cost of microalgal sterol and squalene production can be lowered as a result of the ability to overproduce these lipids. The ability to withstand oxidative stress induced by environmental conditions such as fluctuating light or contaminants in water in this mutant will result in better growth and higher biomass yield. Furthermore, the production of TAG for biofuels was also higher in this mutant. The presence of terbinafine can be used to induce squalene accumulation and reduce contamination. Therefore, several growth conditions can be applied to maximize the levels of desirable products.

## 3. Materials and Methods

### 3.1. Culture Conditions and Mutant Isolation

*C. reinhardtii* wild-type 4A+ strain was provided by Prof. Krishna Niyogi (University of California, Berkeley). Exponential-phase cells at a density of 5 × 10^6^ cells mL^−1^ were diluted to 2 × 10^6^ cells mL^−1^ in fresh Tris-acetate-phosphate (TAP) medium or TAP-N medium as nitrogen-replete or nitrogen-deplete conditions, respectively. Cultures were placed on an orbital shaker at 120 rpm, 25 °C with constant illumination at 50 μmol photons m^−2^ s^−1^. Terbinafine, NaCl, or Rose Bengal was added to warm TAP agar to prepare plates containing each chemical. For terbinafine treatment, terbinafine was added to the culture to a final concentration of 10 μM. Cells were harvested by centrifugation at 6000 rpm for 10 min and kept at −80 °C until use.

To generate a mutant population, culture of *C. reinhardtii* at a density of 5 × 10^6^ cells mL^−1^ was placed in a Petri dish and kept in darkness for 1 h. The culture was irradiated with UV light for 30 min using a UV transilluminator (Model M-26, Upland, CA, USA). This treatment resulted in a 0.1–0.2% survival rate as previously determined. Following an overnight incubation in the dark, cells were placed onto TAP medium plates and incubated at 30–35 μmol photons m^−2^ s^−1^ at 25 °C. After two weeks, visible colonies were patched onto fresh TAP medium plates. A total of 1,920 colonies were obtained. After the patches turned dark green, they were replica plated onto TAP medium plates containing 0.1–1.0 mM of terbinafine to identify strains that exhibited greater resistance to terbinafine than the parental strain.

### 3.2. Analysis of Sterols, Squalene, and Tag Content

Total lipid was extracted from 8 × 10^7^ cells following the method recommended by [49]. Cells were mixed with 4 mL chloroform:methanol (2:1, *v/v*) and shaken until the biomass was dispersed in the solvent system [50]. One milliliter of 0.9% NaCl was added and the tube was vortexed to mix. After centrifugation, a pasture pipette was used to collect the bottom layer and place the liquid into a new tube. It was then dried under nitrogen gas and kept at −80 °C until use.

Squalene was quantified by high-performance liquid chromatography (HPLC). Total lipid was dissolved in 250 μL of acetonitrile and filtered through a 0.2 μm PTFE filter. HPLC was performed using the Waters HPLC system and a C18 column (150 × 3.0 mm, 5 μm particle size, Phenomenex, CA, USA). The mobile phase was 100% acetonitrile with a flow rate of 1.5 mL min^−1^. Squalene was detected at 195 nm and was quantified using a previously generated standard curve of peak area vs. known squalene amount. Sterol content was determined as previously described [29,51]. The sample was analyzed using gas chromatography–mass spectrometry (GCMS-QP20202; Shimadzu, Kyoto, Japan) equipped with a DB-5MS capillary column (Agilent Technologies, Santa Clara, CA, USA), carrier gas: He (1 mL min^−1^); oven temperature: 150–300 °C (increase rate 20 °C min^−1^). The ionization voltage was 70 eV, and the scan range was 40–500 Da. The ergosterol and 7-dehydroporiferaseterol peaks were identified by their respective standards (Sigma-Aldrich, Munich, Germany), and the contents were compared by peak areas.

A relative level of triacylglycerol was quantified using Nile Red fluorescence [52]. In brief, cells were diluted to a density of 2 × 10^6^ cells mL^−1^. Then, 50 μL of dimethyl sulfoxide and 2.5 μL of Nile Red were added to a microtube containing 950 μL of culture. Samples were gently mixed and kept in the dark for 10 min. Two-hundred microliters of each sample was transferred to a 96-well plate. Fluorescent intensity was measured in arbitrary units by a Microplate Reader (TECAN SparkControl v1.1.13.0, Zurich, Switzerland) using the excitation/emission wavelength at 528/576 nm.

### 3.3. Growth, Pigment Quantification, and Photosynthetic Parameter

Cell growth was determined by counting cells on a hemacytometer under a microscope. The photosynthetic pigments were extracted from 1 mL of culture using 1 mL of 80% acetone. The quantity of chlorophyll and carotenoid was calculated following a previously reported formula [53]. The photosynthesis efficiency expressed by the ratio of F_v_/F_m_ was determined using AquaPen AP100 (Photon systems instrument, Drasov, Czech Republic).

## 4. Conclusions

Resistance to terbinafine in green microalgae such as *Chlamydomonas* as noted in this work, can lead to an overaccumulation of sterols and squalene. It is possible to isolate those mutants that also accumulate other high value products such as pigments and TAG. Tolerance to environmental stresses in such strains allows better photosynthetic efficiency and growth performance. If needed, the presence of terbinafine can help eliminate unwanted contamination. Our data offer a novel solution for improving productivity of high value lipids in green microalgae.

## Data Availability

Not applicable.
